# Validation of the Turkish version of the quality of recovery-40 questionnaire

**DOI:** 10.1186/1477-7525-12-8

**Published:** 2014-01-15

**Authors:** Serkan Karaman, Semih Arici, Serkan Dogru, Tugba Karaman, Hakan Tapar, Ziya Kaya, Mustafa Suren, Mehtap Gurler Balta

**Affiliations:** 1 Department of Anesthesiology and Reanimation, Gaziosmanpasa University School of Medicine, Tokat 60100, Turkey

**Keywords:** Turkey, Population, Quality of life, Validation studies

## Abstract

**Background:**

The Quality of Recovery-40 questionnaire (QoR-40) is a self-rated questionnaire used to assess the postoperative recovery quality and health status of patients in the early stages following surgery; however, there is no Turkish version of the QoR-40. The aim of this study was to assess the reliability, validity, and responsiveness of the Turkish version of the QoR-40 (QoR-40 T).

**Methods:**

After the approval of the ethics committee, a total of 137 patients completed the questionnaire during the preoperative period, on the third day, and one month after surgery. The quality of life was evaluated by using a health-related quality of life questionnaire (Short-Form Health Survey-36; SF-36) on the third day and one month after surgery. Reliability, feasibility, and validity were assessed to validate the QoR-40 T.

**Results:**

The Cronbach’s alpha of the global QoR-40 T on the third day after surgery was 0.936. A positive moderate correlation was obtained between the physical comfort, emotional state, physical independence, and pain dimensions of the QoR-40 T and the physical component summary, mental health, physical functioning, and bodily pain subscales of the SF-36 on the third day after surgery, respectively (physical comfort - physical component summary, ρ = 0.292, p = 0.001; emotional state - mental health, ρ = 0.252, p = 0.003; physical independence - physical functioning, ρ = 0.340, p < 0.01; pain - bodily pain, ρ = 0.381, p < 0.01). The standardized responsive mean of the total QoR-40 T was 0.62.

**Conclusions:**

The QoR-40 T showed satisfactory reliability and validity in evaluating the quality of recovery after surgery in the Turkish population.

## Background

Currently, quality is a concept of growing interest in anesthesia, and recovery quality after anesthesia has been assessed as an important measure for evaluating the early postoperative health status of patients [1,2]. The quality of recovery score (QoR-40), which was defined by Myles et al. in 2000, is a self-rated questionnaire consisting of 40 items. It comprises five subscales: comfort, emotion, patient support, physical independence, and pain [1].

The QoR-40 score has been applied in anesthesia and in general, neurological, cardiac, and knee surgery [3-7]. Furthermore, the 36-Item Short Form Health Survey (SF-36), which is a general, health-related questionnaire, has a multidimensional structure that represents most physical and mental aspects of the patient [8-10]. Various studies have revealed that the QoR-40 score showed a substantial correlation with the SF-36 score in patients after cardiac surgery [3,4]. Moreover, Bost et al. demonstrated that the 8-Item Short-Form Health Survey (SF-8), used with a QoR-40 questionnaire to assess the postoperative health status in patients after knee surgery, yielded a satisfactory psychometric evaluation [7].

The QoR-40 can be accepted as an adequate tool to elucidate the impacts of anesthesia and surgery on postoperative recovery and quality of life [11]. However, a systematic search of the literature revealed the absence of a Turkish version of the QoR-40 questionnaire (QoR-40 T) in clinical use. The current study was conducted to evaluate the reliability, validity, and responsiveness of the Turkish adapted version of the QoR-40.

## Methods

### Patients

After the approval of the Gaziosmanpasa University Medical School Ethics Committee (13 − KAEK − 093), patients were informed about the study and written consents for participation were obtained. Patients who were admitted to the outpatient anesthesia unit for preoperative examinations associated with a scheduled elective surgery in general, orthopedic, and otorhinolaryngological surgery under general anesthesia, from April 2013 to July 2013, were included in the study. Those under 18 or over 80 years of age, as well as those who had an alcohol or substance addiction problem, refused to participate in the study, had an American Society of Anesthesiologists (ASA) score of III or IV, or were non-cooperative were excluded from the study.

### The quality of recovery score

The QoR-40 is a self-rated questionnaire that consists of 40 items to evaluate the pain levels and physical and emotional state of patients, comprising five subscales: emotional state (n = 9), physical comfort (n = 12), patient support (n = 7), physical independence (n = 5), and pain (n = 7). Each item is scored on a five-point Likert scale ranging from 1 to 5. The scores for the subscales are given by the sum of the corresponding items, and the total score is computed by a summation of all items. The total score ranges from 40 to 200. In order to design the QoR-40 T, we first obtained development authorization from the original author.

The original version of the QoR-40 was translated from English to Turkish by three individuals, including a native English speaker (university graduate, living in Turkey for 3 years) and two academics from the Department of English Language. The Turkish version of the QoR-40 was back-translated into English by two different English linguistic academicians from the Department of English Language. Thereafter, each question was rendered in its most comprehensible form by a committee of three people which consisted of one health professional fluent in English, a Turkish linguist, and an English linguist.

### Pilot testing

After obtaining the approval of the original author for the backward translation, a pilot test was conducted on 10 randomly selected patients. The pilot testing revealed that all items in the questionnaire were understandable to Turkish patients. With this result, the Turkish version of the QoR-40 was finalized.

### Procedure

Patient characteristics, including age, gender, marital status, and education, were recorded. To evaluate the health status, the QoR-40 T was used during the preoperative period, on the third day after surgery, and one month after surgery. The post-discharge quality of life from the third day and one month after surgery were assessed using the Turkish version of the SF-36 questionnaire. The SF-36 consists of 36 items and standardized response choices, organized into eight multi-item scales, including physical functioning (PF), role limitations due to physical health problems (RP), bodily pain (BP), general health perceptions (GH), vitality (VT), social functioning (SF), role limitations due to emotional problems (RE), and general mental health (MH). It is summarized in two component summary scores, the Physical Component Summary (PCS) and the Mental Component Summary (MCS). All subscale scores range from 0 to 100, with higher scores indicating higher levels of functioning or well-being.

The Kaiser-Meyer-Olkin measure of sampling adequacy and Bartlett’s test of sphericity were used to confirm the assessment of the factor analysis for these items. A principal components analysis was conducted for the construct validity of the QoR-40 T. For the basis of this analysis, the components were extracted with an eigen value of greater than 1. Varimax rotation with Kaiser normalization was selected, and the rotation converged in 7 iterations, with values below 0.40 being suppressed. Additionally, the correlation between the QoR-40 T score on the third-day and the duration of hospitalization, and the QoR-40 T score on the third-day between men and women, were compared to support the construct validity. The internal consistency of the QoR-40 T was also evaluated by using Cronbach’s alpha (α) for the global QoR-40 T and subscale scores. A Cronbach’s alpha coefficient of 0.70 to 0.95 was considered to be acceptable [12]. Item-total correlation coefficients were used to assess the association between the scores. To measure the test-retest reliability, the QoR-40 T was performed in the same patients on the third day and one month after their operations. The postoperative third-day total QoR-40 T score was compared with the recovery visual analog scale (VAS) to assess the concurrent validity (Additional file 1). In addition, predictive validity was determined by assessing the association between the related dimensions of the questionnaires: physical comfort, emotional state, physical independence, pain dimensions of the QoR-40 T and the physical component summary, mental health, physical functioning, and the bodily pain subscales of the SF-36, respectively. This was evaluated by using the Spearman correlation coefficients for each measurement period.

Responsiveness, or sensitivity to clinical change, was measured by evaluating the standardized response mean (SRM). The SRM is calculated by dividing the mean change in the score by the standard deviation (SD) of the change [13,14].

### Statistical analysis

Normality and variance were tested using the One-Sample Kolmogorov-Smirnov, skewness and kurtosis, histograms, and Q-Q plots for each variable. Quantitative data were presented as the means and standard deviation, and qualitative data as the frequency and percentage. Associations were performed by using the Spearman rho correlation coefficient (ρ). Analyses were completed by using the Statistical Package for Social Sciences (SPSS Inc., Chicago, IL) version 20.0 program. The statistical significance for all analyses was set at p < 0.05.

## Results

### Demographic data

Patient characteristics are presented in Table 1. Of the 180 patients approached in the present study, there were 16 refusals (recruitment rate = 0.91); another 27 patients which had been discharged from the hospital could not be reached to complete their questionnaires (completion and return rate = 85%). Thereafter, a total of 137 patients were accepted to participate in the study and completed the questionnaires. The SF-36 was administered to all patients one month after surgery, and all of them completed it. Most of the patients were able to complete the QoR-40 T questionnaire form without any assistance. The mean duration of hospitalization was 3.93 ± 1.29 (IQR 3–5 days).

**Table 1:** Demograhic data

		**Value**
**Age (years)**		41 (18–79)
**Gender (n)**	**Male**	64
	**Female**	73
**Marital status (n)**	**Single**	26
	**Married**	111
**Education (n)**	**Primary school**	69
	**Secondary school**	17
	**High school**	31
	**University**	20

### Reliability and validity

The Cronbach’s alpha (α) values and the Spearman rho (ρ) correlation coefficients of physical comfort, emotion, physical independence, patient support, and pain, with the global QoR-40 score on the third day after surgery, were 0.902, 0.858; 0.878, 0.769; 0.858, 0.603; 0.926, 0.607; and 0.825, 0.680; respectively (Table 2). In addition, the Cronbach’s alpha of the global QoR-40 T on the third day after surgery was 0.936. The item-total correlation coefficients on the third day after surgery ranged from 0.299 to 0.687. Test-retest reliability (Spearman’s correlation coefficient) of the QoR-40 T three days after surgery was 0.658 (p < 0.01). A positive moderate correlation was obtained between the related subscales of the QoR-40 T and SF-36 on the third day and one month after surgery. The physical comfort, emotional state, physical independence, and pain dimensions of the QoR-40 T appeared to be related to the physical component summary, mental health, physical functioning, and bodily pain subscales of the SF-36, respectively (Table 3). Moreover, a positive significant correlation was observed between the total QoR-40 T score and that on the third day after surgery, and the recovery VAS (ρ = 0.468, p < 0.01).

**Table 2:** Correlation coefficients of QoR-40 T and subscales (third day after surgery)

	**T-QoR-40 T**	**PC**	**ES**	**PS**	**PI**	**P**
**PC**	0.858	—	—	—	—	—
**ES**	0.769	0.615	—	—	—	—
**PS**	0.607	0.368	0.466		—	—
**PI**	0.603	0.356	0.273	0.419	—	—
**P**	0.680	0.512	0.423	0.328	0.310	—

Bivariate Spearman’s rho correlation coefficients; all correlation comparisons showed a significance level of P < 0.01.

**T-QoR-40 T:** Turkish version of Quality of Recovery-40 questionnaire. **PC:** Physical Comfort. **ES:** Emotional State. **PS:** Patient Support. **PI:** Physical Independence. **P:** Pain.

**Table 3:** The correlation between the QoR-40 dimensions with the related subscales of SF −36

**QoR-40 T subscale - SF-36 subscale**	**Third day after surgery (Spearman’s rho/p)**	**One month after surgery (Spearman’s rho/p)**
**Physical comfort - physical component summary**	0.292/0.001*	0.512/< 0.01*
**Emotional state - mental health**	0.252/0.003*	0.300/< 0.01*
**Physical independence - physical functioning**	0.340/< 0.01*	0.348/< 0.01*
**Pain - bodily pain**	0.381/< 0.01*	0.226/0.008*

*p < 0.05.

The mean preoperative QoR-40 T was 182 (with an SD of 10.4), and after surgery it was 171 (with an SD of 20). Changes in the QoR-40 T and the subscales before and three days after surgery are presented in Table 4. The mean total QoR-40 T score before surgery was 182 ± 10.4, 171 ± 20 on the third day after surgery, and 186 ± 14 one month after surgery. An SRM of 0.62 was conducted between the preoperative and third day QoR-40 T mean scores. An SRM of 0.2 indicated a small effect of intervention, 0.5 a moderate effect, and 0.8 or greater a large effect [12,13].

**Table 4:** Changes in QoR-40 T and subscales before and 3 days after surgery

	**Maximum possible score**	**Preoperative score Mean (SD)**	**Postoperative score Mean (SD)**	**Mean change (95% CI)**	**SRM**
**T-QoR-40 T**	200	182 (10.4)	171 (20)	−11 (−8 to −14)	0.62
**PC**	60	55 (4.2)	50 (7.9)	−4.5 (−3.2 to −5.7)	0.59
**ES**	45	40 (3.7)	38 (5.1)	−1.6 (−0.9 to −2.4)	0.36
**PI**	25	24 (2.1)	20 (4.9)	−2.6 (−1.8 to −3.4)	0.55
**PS**	35	32 (3)	31 (5)	−1 (−0.1 to −1.9)	0.19
**P**	35	31 (3.2)	30 (4.4)	−1.2 (−0.6 to −1.8)	0.32

**T-QoR-40 T:** Turkish version of Quality of Recovery-40 questionnaire. **PC:** Physical Comfort. **ES:** Emotional State. **PI:** Physical Independence. **PS:** Patient Support. **P:** Pain. **CI:** Confidence Interval. **SRM:** Standardised Response Mean.

The findings of the principal components analysis revealed a five-factor structure in the QoR-40 T of physical comfort, emotion, physical independence, patient support, and pain, as in Myles et al. [1]. Additionally, the ratio of the explanatory importance of the factors by one factor (R^2^) was 0.268. The item loadings in the physical comfort component ranged from 0.532 to 0.804, the emotion component from 0.474 to 0.789, physical independence from 0.561 to 0.871, patient support from 0.620 to 0.845, and pain from 0.541 to 0.776 (Table 5). In addition, the construct validity was supported by a negative correlation with the duration of hospitalization (ρ = 0.265, p = 0.002), and a lower mean postoperative QoR-40 T score was seen in women (167.73 with an SD of 19.90) compared to men (175.03 with an SD of 19.72) (p = 0.015), as indicated by Myles et al. [1].

**Table 5:** Principal components analysis

**Items**	**Mean**	**SD**	**Factor loading**
**Physical comfort**			
I have been able to breathe easily	4.39	0.74	0.532
I have been able to sleep well	3.73	1.09	0.585
I have been able to enjoy food	4.08	1.06	0.553
I have been feeling rested	3.62	1.18	0.627
Nausea	4.13	0.96	0.754
Vomiting	4.29	0.94	0.804
Dry-retching	4.37	0.98	0.776
Feeling restless	4.14	0.96	0.582
Shaking or twitching	4.41	0.88	0.672
Shivering	4.40	0.80	0.592
Feeling too cold	4.37	0.77	0.580
Feeling dizzy	4.23	0.86	0.568
**Emotional state**			
I have been having a feeling of general well-being	4.12	0.76	0.474
I have been feeling in control	4.21	0.85	0.530
I have been feeling comfortable	4.22	0.79	0.562
Had bad dream	4.31	0.76	0.749
Feeling anxious	4.27	0.76	0.745
Feeling angry	4.35	0.72	0.789
Feeling depressed	4.43	0.75	0.769
Feeling alone	4.52	0.77	0.744
Had difficulty falling asleep	4.01	1.03	0.513
**Patient support**			
I have been able to communicate with hospital staff in the hospital	4.43	0.90	0.714
I have been able to communicate with my family and friends	4.63	0.78	0.716
I have been getting support from the doctors in the hospital	4.40	0.92	0.840
I have been getting support from the nurses in the hospital	4.43	0.99	0.845
I have been getting support from my family and friends	4.64	0.80	0.793
I have been able to understand the instructions and advice	4.63	0.79	0.799
Feeling confused	4.21	0.79	0.620
**Physical independence**			
I have been having normal speech	4.27	1.04	0.793
I have been able to wash my face, brush my teeth or shave	4.25	1.24	0.871
I have been able to look after my own appearance	4.32	1.13	0.812
I have been able to write	4.25	1.26	0.561
I have been able to return to work or usual home activities	3.83	1.44	0.651
**Pain**			
Moderate pain	3.63	0.96	0.602
Severe pain	4.31	0.83	0.541
Headache	4.29	0.85	0.682
Muscle pains	4.33	0.99	0.776
Backache	4.32	1.00	0.725
Sore throat	4.45	0.89	0.657
Sore mouth	4.70	0.71	0.603

Kaiser-Meyer-Olkin measure of sampling adequacy (0.832). Bartlett’s test of sphericity (P < 0.01).

Extraction method: Principal components analysis. Varimax rotation with Kaiser normalization; rotation converged in seven iterations and values below 0.40 were suppressed. This model explained 58.91% of the variance.

## Discussion

This study was conducted to evaluate the QoR-40 T, an adapted version of the QoR-40. The QoR-40 T revealed acceptable levels of validity, reliability, and clinical feasibility in the Turkish population.

The principal components analysis showed that the QoR-40 T has the same dimensions indicated by Myles et al. [1]. The results of the factor analysis revealed that our validation study fulfills the requirements of construct validity. In addition, it is known that women usually have a poorer quality of recovery on the basis of previous studies, which was also found in the present study, and supported its construct validity [1]. The current study was able to demonstrate a negative correlation between the QoR-40 T and duration of hospitalization, and psychometric tests have supported the validity and reliability of the QoR-40 T. The predictive validity evaluation revealed a positive correlation between the total QoR-40 T and the subscales of the SF-36. Furthermore, the QoR-40 T showed a satisfactory correlation with the recovery VAS, which was associated with the concurrent validity.

The SRM value of the total QoR-40 T was 0.62, which was previously mentioned as moderate, and indicated a moderate capability for assessing the alterations in the postoperative recovery period [13]. The reliability was confirmed by detecting a Cronbach’s α value of 0.936, which surpassed the recommended level [12]. These findings suggested that the QoR-40 T may, to a reliable degree, be able to evaluate patient recovery.

This study has several limitations. First, there have been few Turkish-validated quality of life assessment questionnaires; therefore, the SF-36 questionnaire had to be used to validate the QoR-40 T. However, patient support, the subscale of the QoR-40, does not have an equivalent or similar scale in the SF-36 or any cross-culturally validated questionnaire, and could not be assessed and validated. Second, associated with the variability of the discharge periods in the surgical clinics and limited sources with which to contact the patients, the time between test and re-test for the QoR-40 T had to be long. Third, patients who underwent elective general surgery, orthopedic surgery, and otorhinolaryngological surgery were included in this study. Therefore, the feasibility of the questionnaire in the general population was potentially restricted. However, this limitation can be overcome by assessing the questionnaire with a larger number of patients in various surgical departments.

## Conclusions

This study showed that the cross-cultural adaptation of the QoR-40 was successfully achieved. The Turkish version of the QoR-40 is a satisfactory, valid, and reliable instrument for clinicians to assess the recovery quality in Turkish patients after surgery.

## Abbreviations

QoR-40: Quality of recovery-40 questionnaire; QoR-40 T: Turkish version of the QoR-40; SF-36: 36-Item short form health survey; PF: Physical functioning; RP: Role limitations due to physical health problems; BP: Bodily pain; GH: General health perceptions; VT: Vitality; SF: Social functioning; RE: Role limitations due to emotional problems; MH: General mental health; VAS: Recovery visual analog scale; SRM: Standardized response mean; SD: Standard deviation; ρ: Spearman rho correlation coefficient; α: Cronbach’s alpha.

## Competing interests

The authors have no competing interests.

## Authors’ contributions

SK designed and collected the study population of the study, SA carried out the data collection, SD paticipated in the design, helped to draft the manuscript and performed the statistical anlysis, TK helped data collection, HT participated in its design, ZK participated in the design, MS helped in the design, and MGB helped data collection. All authors read and approve the final manuscript.

## Supplementary Material

Additional file 1 Visual analog scale. Click here for file
