# Supplementary material for: Validation of the Turkish version of the quality of recovery-40 questionnaire
Source: Health Qual Life Outcomes. 2014 Jan 15;12:8. doi: 10.1186/1477-7525-12-8 (PMC3896711; doi:10.1186/1477-7525-12-8)
Supplement: Additional file 1 — Visual analog scale. [file 1477-7525-12-8-S1.doc]

**Additional file 1.** Visual Analog Scale

Please indicate your condition with a mark “🞨”:

Severe pain No pain

Nausea-vomitting No nausea-vomiting

Immobilised Active
